# Molecular characterization of the A52 murine hepatocellular carcinoma cell line

**DOI:** 10.1002/ame2.70152

**Published:** 2026-02-27

**Authors:** Rhys Gillman, Eun Jin Sun, Miriam Wankell, Matt A. Field, Ulf Schmitz, Lionel Hebbard

**Affiliations:** ^1^ Department of Biomedical Sciences and Molecular and Cell Biology, College of Medicine and Dentistry, College of Science and Engineering James Cook University Townsville Queensland Australia; ^2^ Centre for Tropical Bioinformatics and Molecular Biology James Cook University Cairns Queensland Australia; ^3^ Australian Institute for Tropical Health and Medicine Townsville Queensland Australia; ^4^ Immunogenomics Lab Garvan Institute of Medical Research Darlinghurst New South Wales Australia; ^5^ Menzies School of Health Research Charles Darwin University Darwin Northern Territory Australia; ^6^ Faculty of Medicine & Health The University of Sydney Camperdown New South Wales Australia; ^7^ Storr Liver Centre, Westmead Institute for Medical Research Westmead Hospital and University of Sydney Sydney New South Wales Australia; ^8^ Present address: Discipline of Molecular and Cell Biology Townsville QLD 4811 Australia

**Keywords:** carcinoma, cell line, gene expression profiling, genomics, hepatocellular, mice

## Abstract

**Background:**

To combat hepatocellular carcinoma (HCC) disease heterogeneity and growing mortality, there is an urgent need for targeted and personalized therapeutics. While syngeneic mouse models are commonly used for preclinical validation of these therapeutics, the lack of genetically characterized murine cell lines adds uncertainty to the study of drug‐gene interactions in these models. We previously generated a novel murine cell line, A52, from a diethylnitrosamine (DEN)‐induced adiponectin‐knockout mouse model. Here, we present a comprehensive genomic and transcriptomic characterization of the A52 cell line.

**Methods:**

A52 cells were grown in various culture medium compositions to investigate robustness to simple media. Whole‐genome sequencing (WGS) and RNAseq were performed on A52 cells from both cell culture and syngeneic tumor tissue, as well as a reference cell line representing non‐tumor cells, AML‐12.

**Results:**

A52 was found to show robust growth in all medium compositions. Substantial chromosomal instability was observed in A52, including trisomy 15 and notable amplifications of oncogenic loci such as *Myc* and *Cd274* (PDL‐1), alongside frequent small variants and structural rearrangements. Notably, the cell line harbors the common HCC driver *Braf* V584E mutation, and a novel *Plk1* p.R364W variant predicted as a driver mutation. Transcriptomic profiling defined a distinct “A52 gene signature” enriched in EGFR‐ERBB signaling and cell migration pathways. Integrative analyses demonstrated that the A52 gene signature aligns closely with a subset of human HCC lacking CTNNB1 mutations.

**Conclusion:**

This study provides a critical genetic resource, facilitating more precise preclinical modeling and therapeutic validation in HCC.

## Graphical abstract

Hepatocellular carcinoma (HCC) is a poor outcome cancer with limited therapeutic choices. To advance therapeutic development we genetically characterized the A52 murine HCC cell line. We noted genetic changes that match a subset of human HCC and this offers the opportunity to test novel targeted therapies in syngeneic mice.

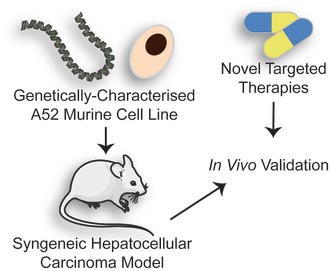

## 1 INTRODUCTION

Liver cancer has the second‐highest mortality rate among cancers globally, which is disproportionately high considering its incidence rate ranks seventh worldwide.^1^ Both incidence and mortality of the disease have been rising rapidly around the world, underscoring the urgent need for improved therapeutic strategies.^2^, ^3^ Hepatocellular carcinoma (HCC), the most common form of liver cancer, notably lacks effective treatments, largely due to significant tumor heterogeneity.^4^ This complexity necessitates the development of targeted and personalized therapies that are tailored to individual patients' mutational profiles, although substantial challenges remain in developing these approaches.^5^

Targeted therapies comprise a class of drugs that act upon specific proteins or even protein alterations and are the cornerstone of precision therapies. However, the specificity of these therapies significantly complicates preclinical validation processes. After their initial identification, in vivo validation of targeted therapies is crucial to understand off‐target effects and drug interactions within the tumor microenvironment. Furthermore, the efficacy of even non‐immune acting small molecule inhibitors can be influenced by the immune system, such as by improving immune cell infiltration, reversing immunosuppressive activities of immune cells, or by directly affecting immune cells.^6^ Thus, the shift from traditional cytotoxic therapies to targeted therapies necessitates effective in vivo models with clearly defined genetic backgrounds.

Several mouse models of HCC are used for preclinical studies. These include, endogenous models induced with diethylnitrosamine (DEN), metabolic‐associated fatty liver disease (MAFLD), and alcohol mimic natural HCC development, but they are slow to develop and provide limited or no control over cancer mutations. Alternatively, genetically engineered models allow ultimate control of genetics, but are also slow and complex to produce. Transplantation models, on the other hand, where tumor cells are injected into mice, have emerged as a practical solution due to their simplicity, speed, and ease of genetic manipulation, facilitating specific drug‐gene interaction studies.^7^ For humanized models, human cell lines are used in immunodeficient nude mice,^8^, ^9^ but these models then ignore immune functionality, a key player in HCC progression. Thus, syngeneic models have become preferred, in which mouse HCC cells are injected into genetically alike mice, controlling the cancer genotype, and avoiding the need for immunodeficiency,^10^, ^11^ and hence making them important for in vivo validation of targeted therapies. Despite their frequent use, no murine HCC cell line has yet been thoroughly genetically characterized, leaving studies vulnerable to unknown genetic confounders.

Hence, for the purpose of targeted drug validation, it is vital to know what background mutations are present in the cell line and how this could affect drug‐response. By addressing this critical gap, our study aims to provide comprehensive genomic and transcriptomic characterization of the novel A52 murine HCC cell line. This cell line was derived from a DEN‐treated adiponectin (APN)‐KO mouse model of HCC.^12^, ^13^ We previously demonstrated its use as a syngeneic mouse model, characterized its protein expression and demonstrated consistency with human HCC.^13^ The current study further explores the genetic landscape of the A52 cell line, identifying its potential molecular drivers and characterizing its suitability for preclinical studies targeting specific genetic alterations.

## 2 METHODS

### 2.1 Cell culture

A52 cells were grown on plates pre‐coated with 0.5% porcine gelatin (Merck) for 30 min at 37°C to enhance cell attachment. Cells were maintained at 37°C in a humidified incubator with 5% CO_2_ in Dulbecco's Modified Eagle Medium (DMEM, high glucose, Gibco) supplemented with 20% fetal bovine serum (FBS), 10 μg/mL hydrocortisone, 1 mmol/L sodium phenobarbital, 2 μg/mL bovine insulin, 20 ng/mL human epidermal growth factor (EGF), and 1% penicillin–streptomycin (P/S). AML‐12 (ATCC, #30‐2006) cells were cultured in DMEM:F12medium containing 10% FBS, 10 μg/mL bovine insulin, 5.5 μg/mL transferrin, 5 ng/mL selenium, 40 ng/mL dexamethasone, and 1% P/S (all reagents were purchased from Merck). Cells were grown to 60%–80% confluency prior to RNA extraction.

### 2.2 Live cell imaging

For live‐cell imaging experiments, A52 cells were seeded into gelatin‐coated 96‐well plates at 1000 cells per well. To assess cell attachment dynamics, imaging was performed every 30 min for 17 h using an Incucyte SX1 live‐cell imaging system (Sartorius). For cell growth assays, imaging intervals were set at every 2 h. Each condition was analyzed with 12 replicates. Cellular confluence was quantified using Incucyte Software (version 2023A Rev2) with the AI Confluence segmentation algorithm.

### 2.3 Mouse model

To generate A52 tumors, 2.5 × 10^6^ A52 tumor cells in a volume of 200 μL (1:1 PBS and Matrigel) were injected subcutaneously into the bilateral rumps of C57BL6/J mice (6–12 weeks of age). The mice were checked daily and tumor growth monitored with digital calipers. At the end of the experiment the mice were euthanised with carbon dioxide inhalation and the tumors were removed and snap frozen in liquid nitrogen and stored at −80°C. All procedures were approved by the James Cook University Animal Ethics Committee (A2983).

### 2.4 Whole genome sequencing and RNAseq analysis

Whole genome sequencing (WGS) was performed using Illumina Next‐Generation Sequencing (NGS) on genomic DNA extract from A52 cell lines in culture. Variant analysis was performed using the Genome‐Analysis Toolkit (GATK) (v4.5.0.0)^14^ pipeline, and mutational signatures were extracted using SigProfilerAssignmentR.^15^ Structural variants (SVs) were analyzed using Manta (v1.6.0)^16^ and genome‐wide depth analysis used Samtools (v.1.20).^17^

RNAseq analysis was performed using Illumina NGS on RNA extracted from A52 and AML‐12 cells in duplicate, and from five independent tissue samples of subcutaneous A52 tumors. Additional data were sourced from three healthy mouse liver samples from the RNA mouse atlas,^18^ and 377 human HCC tissue samples and 50 non‐cancer control samples from The Cancer Genome Atlas (TCGA).^19^ Differential gene expression analysis was performed using DESeq2 (v1.42.1)^20^ using thresholds of adjusted *p*‐value <0.01 and log2 fold‐change >1 for significance, and pathway enrichment analysis was performed using TopGO (v2.54.0). Personalized driver gene prediction was undertaken using the TARGET‐SL framework^21^ and DawnRank.^22^ The full list of driver gene predictions is available in Table S1 Driver Genes.

Further detail of the sequencing analyses is provided in the Supporting Information Methods.

## 3 RESULTS

### 3.1 A52 cells grow well in simple medium

**FIGURE 1:**
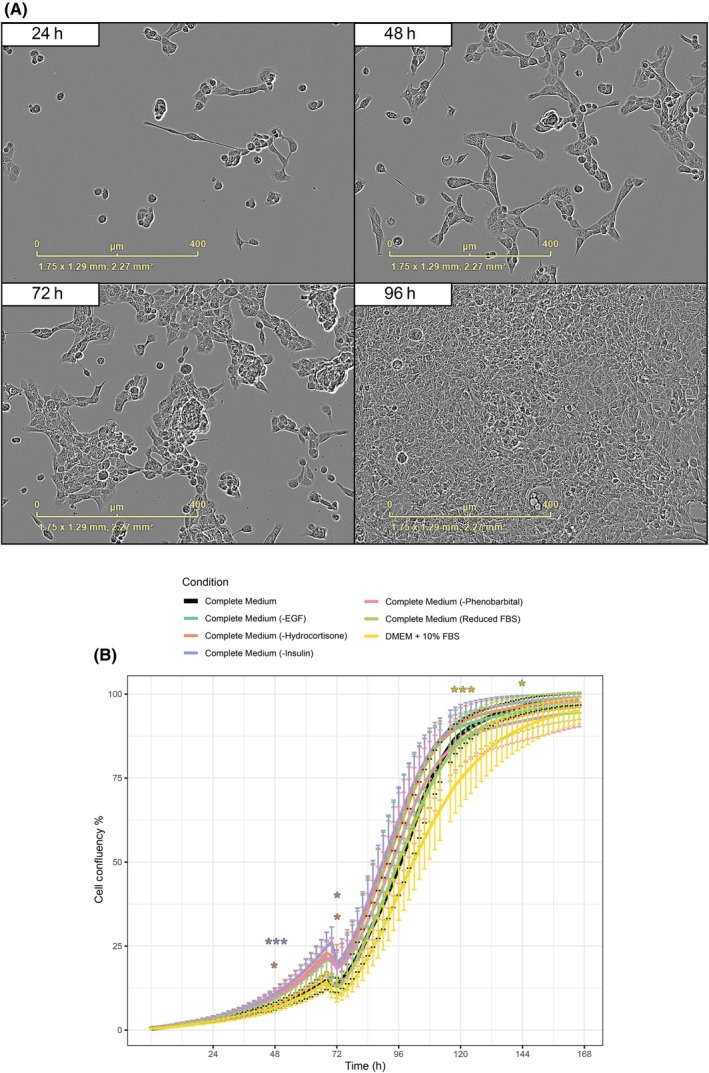
Growth characteristics of A52 cells. (A) A52 cells form an adherent monolayer with epithelial morphology and grow rapidly in 2‐dimensional culture. (B) A52 growth is robust after removal of complex media additives. Cells were grown in 96‐well plates with 12 replicates per condition for 7 days, with medium refreshed after 72 h. Plotted values are mean and standard deviation. Asterisks indicate significant differences from the Complete Medium condition at every 24 h timepoint using one‐way ANOVA and Tukey's post hoc testing and the Benjamini Hochberg correction. *p < 0.05; ***p < 0.001.

The A52 cell line was derived from a DEN‐treated *Adipoq* knockout mouse HCC.^12^, ^13^ The general characteristics of the cell line are presented in Table 1. A52 cells were grown from low confluency and observed under live cell imaging for 7 days. A52 is an adherent cell line with epithelial morphology that attaches best after gelatin coating. The cells form a monolayer in culture, with individual cells approximately 20–30 μm in size (Figure 1A).

**TABLE 1:** General characteristics of the A52 cell line.

Characteristic	
Adherent	Yes
Source	Mouse (*M. musculus*), C57BL/6J
Morphology	Epithelial
Tissue	Liver
Cell type	Hepatocyte
Growth medium	DMEM (high glucose) +20% FBS +10 μg/mL Hydrocortisone +1 mmol/L Na Phenobarbital +2 μg/mL Bovine Insulin +20 ng/mL Human EGF +1% Penicillin Streptomycin Gelatin Coated Plate
Doubling time	18 h
Attachment time	6–12 h

HCC cells do not typically proliferate in in vitro conditions without specific media additives.^23^ For this reason, the A52 cell line has been maintained in a medium specifically designed to allow their expansion. To assess the robustness of A52 cells and their dependency on specific growth factors, we systematically tested cell growth in varying medium compositions for 7 days, with their medium composition altered after 18 h of attachment in complete medium. We observed attachment within 6–12 h based on average confluence measures (Figure 1B). Unexpectedly, we found that A52 cells were highly tolerant to the removal of most medium components; notably, removing phenobarbital and insulin resulted in a small yet significant growth advantage within the first 48 h. Only the simplest medium condition, DMEM +10% FBS with all additives removed showed a slight but significant growth reduction (Figure 1B).

### 3.2 A52 cells harbor many mutations consistent with human HCC

**FIGURE 2:**
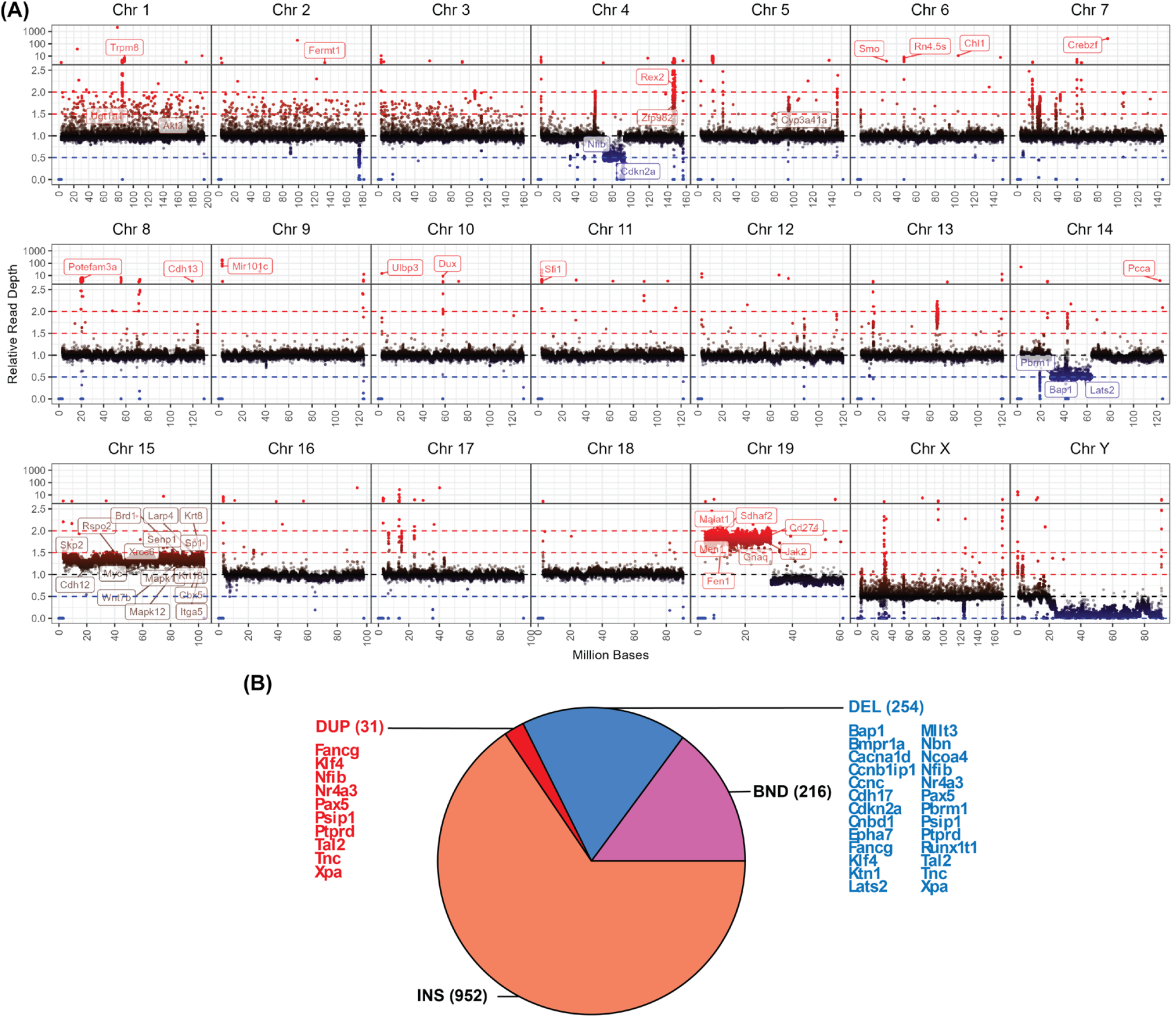
Structural variants in the A52 genome. (A) Read depth, relative to the average per‐base depth, was plotted genome‐wide as the mean for each 10k‐base window. Genes of interest within regions of higher or lower than expected read depth are indicated. (B) Pie chart of structural variant types called using Manta. Variants covering exons of genes in the Cancer Gene Census (CGC) are listed. BND, Breakends; DEL, Deletion; DUP, Duplication.

Genomic DNA was extracted from the cell line and Illumina whole genome sequencing (WGS) was performed. To identify genetic variants in the cell line, we compared the DNA against the GRCm39 reference genome. Given that these cells originated from HCC tissue and have been cultured extensively, rederived from tumors and cultured again, this resulted as expected in the identification of a number of both large‐ and small‐scale genetic alterations. An average per‐base depth of coverage of 60× was achieved. We evaluated genome‐wide sequencing depth relative to the average per‐base depth across all autosomes to identify large chromosomal abnormalities (Figure 2A).

Strikingly, chromosome 15 displayed a consistently elevated read depth, approximately 1.3‐fold greater than the genome‐wide average. This suggests a possible trisomy of chromosome 15; however, given that the depth is lower than the expected 1.5‐fold increase, this may be heterogeneous in the cell population. We noted several well‐known oncogenes on this chromosome including *Myc*, *Rspo2*, *Wnt7b*, *Sp1*, and invasion‐related genes including *Krt8/18*, *Cdh12*, and *Itga5*. Additionally, a large amplification was apparent on chromosome 19 which spans approximately half of the chromosome, containing the *Cd274* gene which encodes the PDL‐1 immune checkpoint protein, as well as other oncogenes such as *Malat1*. The sequencing depth of this amplified chromosome 19 region was up to 2x the average depth, suggesting more than one additional copy of the region. Smaller‐scale amplifications were evident throughout the genome, suggesting several duplication events, including regions containing *Smo* and *Pcca* genes. Additionally, large deletions were observed in chromosomes 4 and 14 spanning several key tumor‐suppressors including *Cdkn2a*, *Pbrm1*, *Bap1*, and *Lats2*. Based on read depth, we were also able to confirm disruption of exon 2 of the *Adipoq* gene, which contains the translation initiation start site (Figure S1).

We further utilized Manta for smaller‐scale SV detection (Figure 2B). After filtering, 1453 SVs were detected, predominantly comprising insertions. SVs were detected spanning exons in 1124 genes, of which 27 were known cancer‐related genes in the Cancer Gene Census (CGC) database.^24^ Several of these were known to be frequently altered in HCC and other cancers including deletions in *Bap1*, *Cdkn2a*, and *Lats2*, as well as duplication of *Ptprd*.^25^, ^26^

**FIGURE 3:**
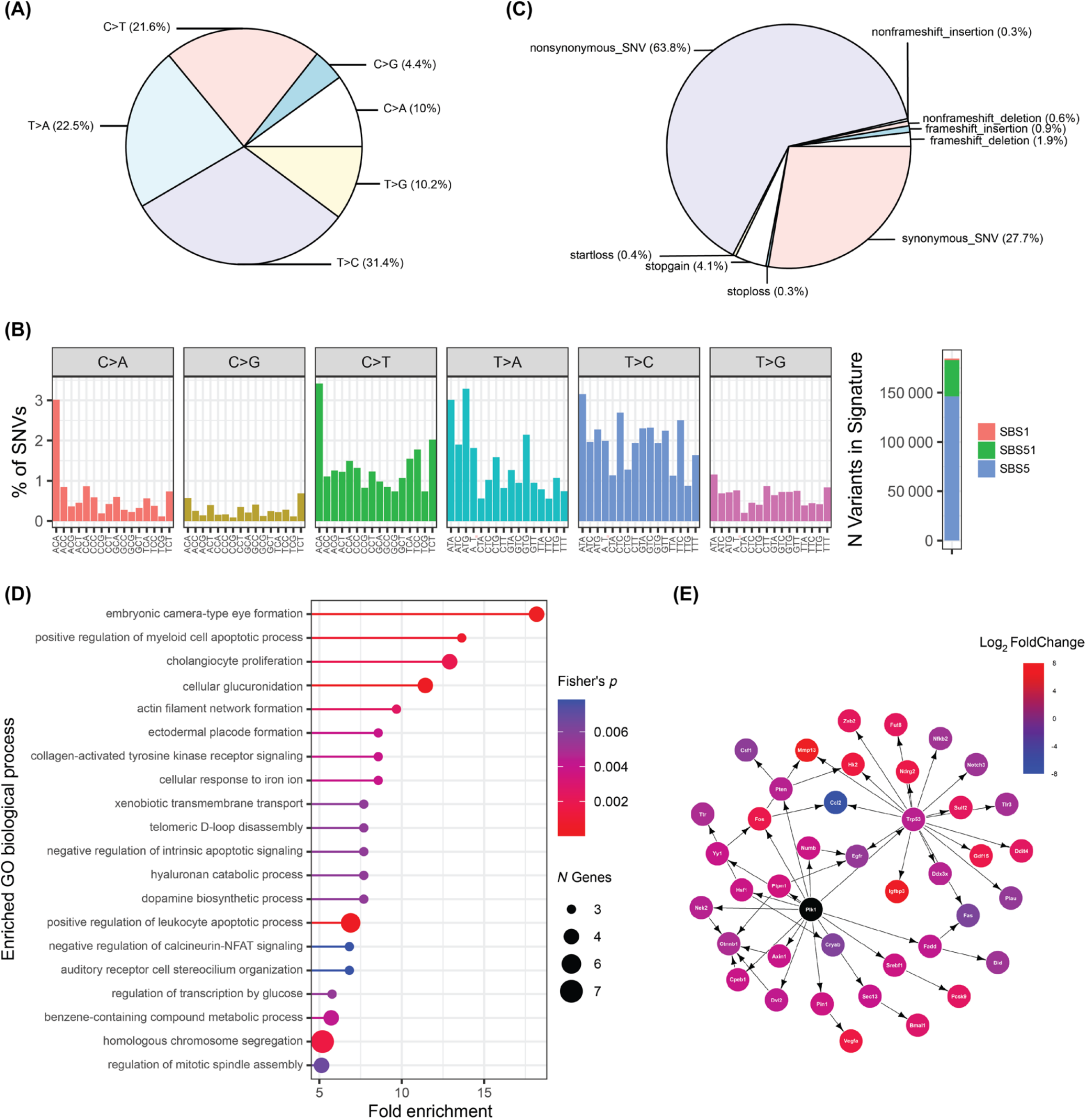
Small variants in the A52 genome. (A) Frequency of base substitutions from the pyrimidine base pair. (B) Left, frequency of base substitutions from the pyrimidine base pair with trinucleotide context following COSMIC SBS96 convention. Right, assignment of variants to COSMIC SBS96 mutation signatures by SigProfilerAssigmentR. (C) Frequency of variant types assigned by ANNOVAR. (D) Gene Ontology (GO) Biological Process enrichment of genes with non‐silent mutations in A52. (E) Gene interaction neighborhood of differentially expressed genes influenced by *Plk1*.

Small variants, including single nucleotide variants (SNVs) and small insertions and deletions (INDELs), were identified following GATK best practices. Firstly, we considered all single base substitution types across the genome. All categories of base substitutions were detected, G>C transversions were rare, and T>A, T>C, and C>T substitutions predominated, consistent with DEN‐induced HCC models.^27^ However, the A52 cell line had a higher‐than‐expected frequency of C>A transversions (Figure 3A). We further investigated the SBS96 trinucelotide context of base substitutions using COSMIC SigProfilerAssignmentR. We found that the mutational signature was relatively consistent with that published,^27^ and the higher frequency of C>A transversions was found to be specific to the ACA trinucleotide context. SigProfilerAssignmentR assigned the highest proportion of mutational signatures to SBS5, a “clock‐like” signature consistent with mutation accumulation over time (Figure 3B).

To further investigate specific small mutations, we focused on exon coding variants to identify variants likely to impact protein function. Small variants consisted predominantly of non‐synonymous and synonymous SNVs, with stop‐gain variants the next most‐frequent, consistent with the DEN HCC model^27^ (Figure 3C). Non‐silent small variants were identified in 1123 genes, enriched in pathways associated with apoptosis, cholangiocyte proliferation, and glucuronidation (Figure 3D). Considering human orthologs of these mutated genes, we identified 61 known HCC driver genes in the CGC database. Notably, we identified the V584E variant in *Braf*, reported as a key driver in DEN‐induced HCC.^27^ The presence of this heterozygous variant was confirmed via Sanger sequencing (Figure S2).

### 3.3 Plk1 is a candidate novel driver gene in A52 cells

Due to the large number of total variants likely consisting of many passenger mutations, we sought to identify driver mutations in the A52 cell line. We used DawnRank^22^ within the TARGET‐SL framework, as published by us,^21^ to predict driver genes by quantifying mutation impact on network‐level gene dysregulation, combining variant information with gene expression. We used the mouse STRINGdb (v11) as a reference network. The highest‐ranking driver prediction in the A52 cell line was *Plk1* (p.R364W), a well‐studied oncogene involved in neoplastic transformation (Figure 3E). We confirmed the presence of this variant via Sanger sequencing (Figure S2). In the context of the STRINGv11 interaction network, this mutation was closely associated with several highly dysregulated cancer‐related genes including *Mmp13*, *Igfbp3*, *Fas* and *Ccl2* (Figure 3E).

### 3.4 A52 cells have altered gene expression in several cancer‐related pathways

**FIGURE 4:**
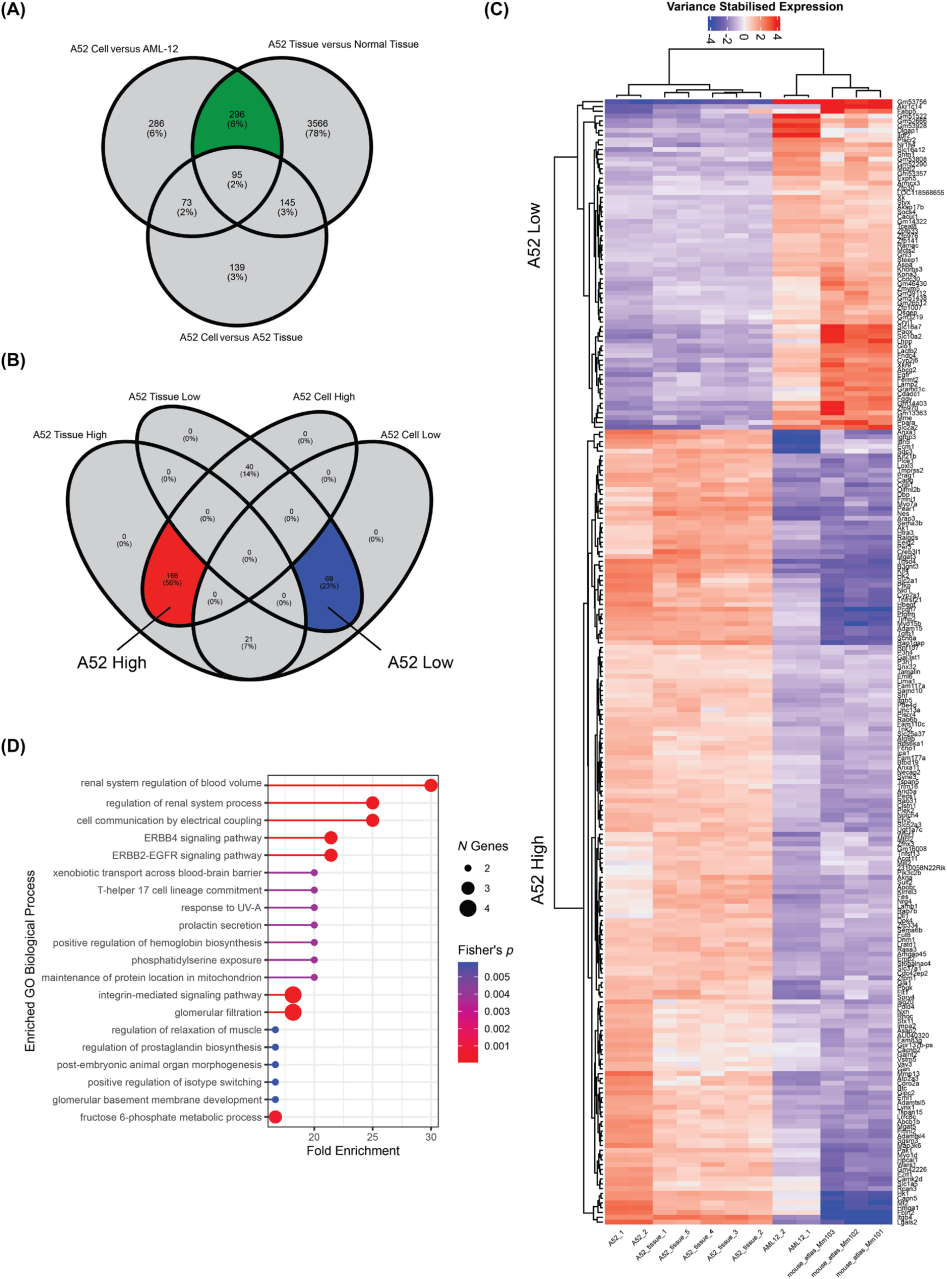
A52 gene expression signature. (A) Pairwise differentially expressed genes (DEGs) were identified using DESeq2, and 296 consistent DEGs found in A52 tissue and A52 cells were selected. (B) The 296 A52 DEGs were further filtered to retain those consistently up‐ or down‐regulated in both A52 tissue and A52 cells, this list is the A52 gene signature. (C) Heatmap of the A52 gene signature in two A52 cell samples, five A52 tissue samples, two AML‐12 immortalized normal hepatocyte samples, and three mouse liver samples from the mouse RNA atlas.^18^ Expression values come from DESeq2 variance stabilizing transformation (VST) centered by the mean of each gene. (D) Gene Ontology (GO) Biological Process enrichment of genes in the A52 gene signature.

Next, we investigated the gene expression of A52 cells and A52 syngeneic tumor in relation to a reference hepatocyte cell line and the normal mouse liver, respectively, to identify pathway dysregulation. We first performed pairwise differential expression analyses between these groups, with the A52 tissue versus normal liver _showing the greatest number of differentially expressed genes (DEGs; *n* = 4102). As expected, we found several DEGs between A52 cells and A52 syngeneic tissue (*n* = 452). To discard these differences and to focus on the gene expression pattern that best describes the A52 cell line, we took the intersection of DEGs unique to A52 cells and tissues (Figure 4A; *n* = 296) and filtered to retain those that were up‐ or down‐regulated in both (Figure 4B). This produced a set of 235 DEGs which we termed the *A52 signature*, which was further divided into 166 A52 High genes and 69 A52 Low genes.

The A52 gene signature included several HCC‐related genes (CGC database) including *Egfr*, *Fes* and *Klf4*, while most others were non‐HCC genes (Figure 4C). Gene ontology analysis of the A52 gene signature found highly significant gene dysregulation in important cancer processes including EGFR‐ERBB2/4 signaling, integrin signaling and cell migration (Figure 4D).

### 3.5 A52 cell gene expression is representative of non‐CTNNB1 mutated human HCC

**FIGURE 5:**
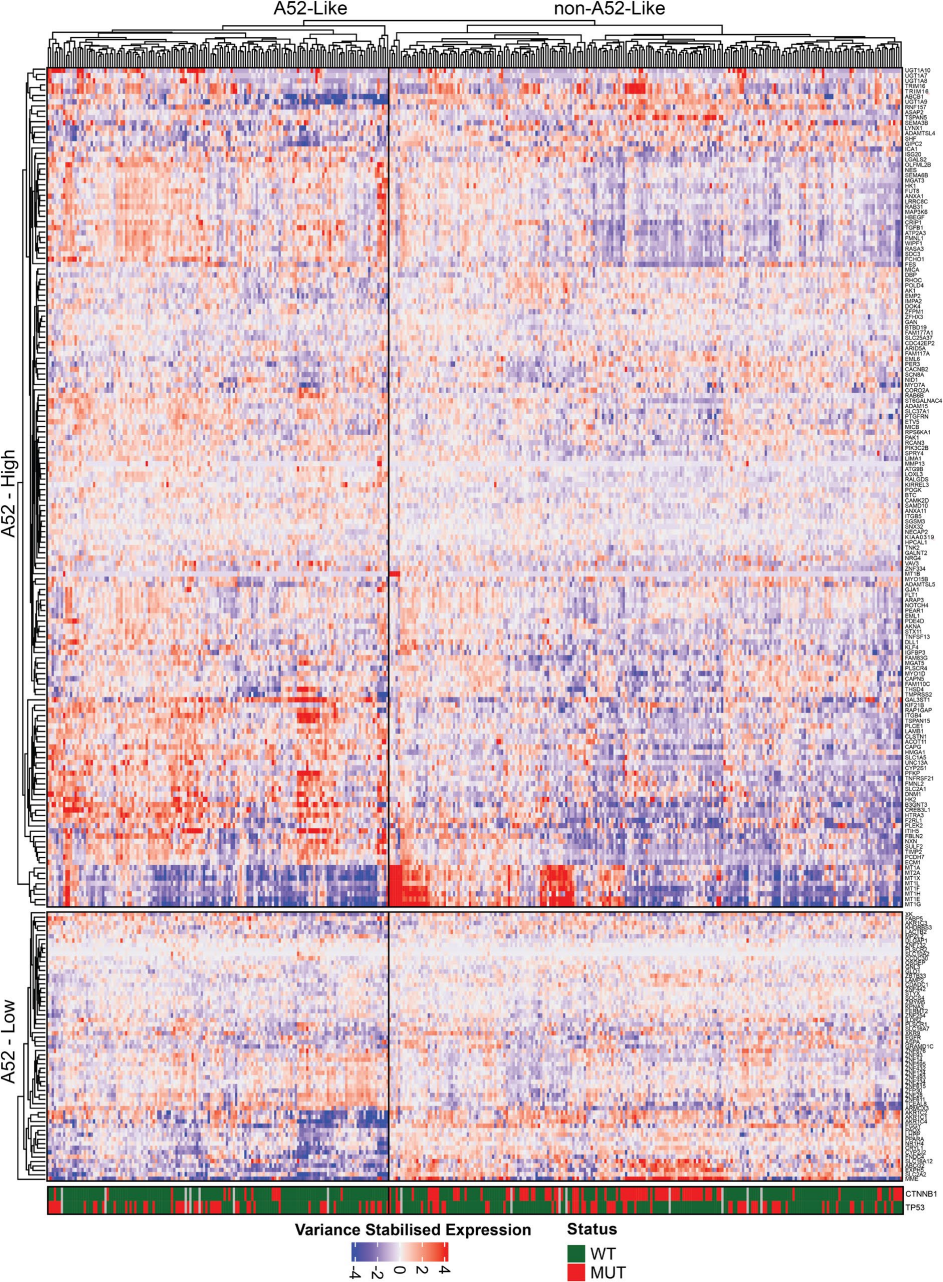
Expression of the A52 gene signature in The Cancer Gene Atlas (TCGA) Liver Hepatocellular Carcinoma (LIHC) patients (*n* = 373). Hierarchical clustering of genes and patients performed by Stats hclust function using the “complete” method and Euclidean distance. The gene expression heatmap is split into A52 high genes (top) and A52 low genes (bottom). The topmost branch of the hierarchical cluster was used to divide patients into A52‐like (*n* = 77) and non‐A52‐like (*n* = 296). The bottom section of the heatmap indicates TCGA LIHC patients with mutant (MUT) or wild‐type (WT) CTNNB1 and TP53.

Finally, we sought to determine whether the A52 gene signature is present in human HCC. We identified human orthologs of the A52 gene signature and investigated their expression in TCGA Liver Hepatocellular Carcinoma (LIHC) patients (Figure 5). Using hierarchical clustering, we identified a cohort of 78 patients (20.9%) with A52‐like gene expression. We investigated differences between A52‐like and non‐A52‐like patients in terms of clinical data and mutations with greater than 5% frequency in the cohort using Chi‐Square testing. A52‐like patients were significantly more likely to lack a *Ctnnb1* mutation (BH‐adjusted *p* = 0.0008), with a frequency of 5.48% compared with 31.03% in non‐A52‐like patients. While non‐significant (BH‐adjusted *p* = 0.09), the frequency of TP53 mutations was also much higher in A52‐like patients (43.84%) compared with non‐A52‐like patients (26.55%). Additionally, A52‐like patients tended to have rarer forms of liver cancer, including mixed hepatocholangiocarcinoma and fibrolamellar carcinoma. There were no other significant differences between these patient groups in terms of clinical information such as survival (Figure S3), tumor stage, risk factors or fibrosis scoring.

## 4 DISCUSSION

HCC remains challenging to treat due to extensive genetic heterogeneity, necessitating the development of targeted and personalized therapies that require reliable preclinical models. Understanding the genetic basis of tumor models is paramount, as it informs therapeutic targeting, efficacy predictions, and translational relevance. Syngeneic transplanted models offer a fast and simple method within a complete immunocompetent mouse model. However, cell lines are notorious for chromosomal instability and accumulating mutations.^28^ The lack of genetically characterized murine HCC cell lines available for use by researchers means that in vivo studies of drug‐gene interactions using these models have countless unknown confounders. Here, we provide the first comprehensive genomic and transcriptomic characterization of the murine HCC cell line A52, highlighting its potential as an accurate preclinical model.

A52 is an epithelial adherent, fast growing cell line, and we demonstrated its robust growth in simple culture medium. Primary HCC cells and hepatocytes fail to proliferate in standard hepatocyte medium, but supplements like phenobarbital and EGF can overcome this.^23^, ^29^ Following these suggestions, A52 has been cultured extensively in high FBS (20%), and with additional insulin, hydrocortisone and EGF. However, we found that A52 cells grown in medium supplemented with only 10% FBS had only slightly impaired growth relative to those in the complex medium, suggesting that the cells did not benefit from the additional growth factors. Additionally, these cells have been cultured with phenobarbital based on previous suggestions,^23^, ^29^ but it offered little to no proliferative advantage and even appeared to inhibit growth. Based on these results, while users of the A52 cell line are welcome to reduce the costs of short‐term functional studies by growing these cells in simple medium, it should be noted that the effects of this have not been rigorously tested over long‐term culture, and we therefore recommend maintaining long‐term stocks in complete medium.

We expanded our earlier in vitro and in vivo work with A52^13^, ^30^ using genomic and transcriptomic sequencing technologies. Unsurprisingly, given that cell lines are characterized by massive chromosomal instability,^28^ the C57B6/J‐derived A52 cells carry numerous genetic variants diverging from the reference genome, including large scale aneuploidy, structural variants, and many small variants, complicating the identification of the key oncogenic drivers. Genome‐wide read‐depth analysis showed evidence for trisomy 15, and major amplification in chromosome 19. Previous studies have reported on frequent gain of chromosomes 15 and 19 in mouse liver neoplasia.^31^, ^32^ Chromosome 15 amplification is frequently attributed to *Myc* (*c‐Myc*), a gene known to play a key role in the immortalisation of cell lines.^33^ However, we also noted several other chromosome 15 oncogenes and invasion‐related genes that may play a role in A52 carcinogenesis. *Myc* is an oncogenic transcription factor that regulates cell proliferation, is frequently amplified and overexpressed in human HCC, and plays a key functional role in hepato‐carcinogenesis.^34^ Notably, mouse chromosome 15 is syntenic with human chromosome 8q and 5p, which together are two of the most frequently amplified regions in human HCC and include the human MYC gene.^26^, ^35^ This suggests that A52 could be valuable for preclinical testing of therapeutics targeting Myc‐driven tumors.

Additionally, in A52 cells, the highly amplified region of chromosome 19 is consistent with previous reports of double minutes (DM) amplifications of chromosome 19 in mouse HCC, characterized by massive amplification of chromosomal fragments.^36^ We identified amplification of *Cd274* (PDL‐1) in this region which is important to note for in vivo immune‐related drug trials. We also observed two large deletions in chromosomes 4 and 14 which appear to be heterozygous. These regions contained tumor‐suppressor genes which could explain their selective pressure, and includes *Cdkn2a*, *Pbrm1*, *Bap1*, and *Lats2*.

The A52 cell line was derived from a DEN‐induced model of hepatocarcinogenesis. DEN is a well‐known tumor‐inducer which causes DNA adducts in the liver, resulting in random mutagenesis.^37^ The mutation profile of A52 showed predominant T>C and T>A substitutions and low C>G substitutions, consistent with DEN‐induced liver mutagenesis^27^, ^38^ and human HCC.^39^ The trinucleotide‐context mutation signature SBS5 was highly represented in A52 cells, which has also been observed in human HCC,^39^ but not in DEN‐induced HCC.^27^ SBS5 is a clock‐like signature associated with cumulative mutations over time,^40^ and is thus frequently observed in cell lines.^41^ The dominance of the SBS5 signature suggests cumulative genomic instability consistent with prolonged in vitro culturing, reinforcing the importance of periodic genomic revalidation in cell‐line‐based research. We did observe higher than expected C>T substitutions, which can be indicative of mismatch repair deficiency,^42^ although the only mismatch repair variant we identified was *Msh5* I72N, which is more frequently associated with meiotic defects. Thus further investigation may be required. Finally, a higher frequency of C>A substitutions is commonly associated with oxidative damage,^43^ which may be due to the adiponectin‐KO background of A52, since adiponectin has been shown to protect against oxidative stress.^44^

DEN‐mice often acquire mutations in any of 4 drivers, namely *Hras, Braf, Egfr* and *Apc*.^27^ We identified the hotspot *Braf* mutation V584E in A52. *Braf* is a regulatory serine/threonine kinase that functions downstream of growth factor receptors to activate the *Mapk/Erk* pathway.^45^ Many protocols introduce long‐term phenobarbital (PB) exposure after DEN to increase the probability of carcinogenesis, which also selects for *Ctnnb1* mutations.^27^, ^46^, ^47^ PB promotes cell proliferation through *Car* (Constitutive androstane Receptor or *NR1I3* Nuclear Receptor Subfamily 1 Group I Member 3) activation,^48^ but also down‐regulates *Ctnnb1* through *Egfr* inhibition, giving cells with constitutively active *Ctnnb1* a selective advantage.^49^ Unexpectedly, we found no *Ctnnb1* mutation in A52, possibly explaining the ineffectiveness of phenobarbital in culture. Given that phenobarbital may inhibit the growth of *Ctnnb1* positive cells due to *Egfr* inhibition, the driver *Braf* mutation which functions downstream of *Egfr* may be responsible for rescuing this effect. Indeed, we observed low expression of the *Egf* receptors *Egfr* and *Erbb2*, but high expression of EGF‐family genes including *Hbegf* and *Btc*, possibly due to long‐term phenobarbital exposure in vitro.

We identified many more mutations across the genome in A52. These variants were enriched in apoptosis and proliferation pathways, suggesting their mutagenesis was non‐random and likely contributes towards A52 tumorigenesis. Using DawnRank,^22^ we predicted de novo driver genes in A52. *Plk1*, carrying a p.R364W variant, was the highest ranked prediction. *Plk1* is another serine/threonine kinase with many down‐stream targets, primarily involved in cell cycle regulation. While *Plk1* overexpression and some variants are well associated with HCC,^50^, ^51^ and *Plk1* has been linked to a specific subtype of human HCC,^52^ the p.R364W variant has not been previously reported.

We assessed A52 gene expression in the context of both in vitro cell culture and in vivo syngeneic tumor tissue, with respective non‐cancer controls. Considering both contexts, we composed an A52 signature of differentially expressed genes that represent A52's cancer‐associated gene dysregulation. This signature was enriched in pathways expected for a liver cancer model, including apoptotic and proliferation pathways. To investigate its suitability as a model for human HCC, we analyzed expression of the A52 gene signature in TCGA LIHC patients. This revealed a subgroup of patients with A52‐like expression, which significantly differed from non‐A52‐like patients due to a lack of *Ctnnb1* mutations. Although we found no significant difference in survival between the A52‐like and non A52‐like patients in the TCGA cohort, *Ctnnb1* mutation status can be considered to constitute a bona fide HCC subclass, with *Ctnnb1* wild‐type patients previously reported to present with poorly differentiated, proliferative tumors and poorer prognosis, as well as characteristic gene expression and metabolic features.^53^ Given that genomic sequencing data is not available for other murine HCC cell lines to confirm genetic features such as this, this underscores the utility and translational potential of A52 to model this distinct subclass of poor prognosis HCC.

The primary goal of this paper was to provide a resource of known genetic variants and pathway dysregulation for the A52 cell line. However, it is also important to understand what genetic alterations are responsible for driving carcinogenesis in the cell line, and to indicate which types of HCC A52 best models. We identified numerous likely driver mutations including a copy‐number amplification of *Myc* and *Cd274*, and small variants in *Braf* and *Plk1*, and, based on their similarity in gene expression, A52 may represent a worthy model of non *Ctnnb1* mutated HCC. Despite comprehensive genomic characterization, functional validation of identified variants was beyond this study's scope. Future studies should investigate individual variants functionally through targeted genetic manipulation to confirm driver roles. Additionally, and as with all widely used cell lines, periodic re‐sequencing is recommended for users of the A52 cell line to ensure this resource remains correct. This publication will provide a key resource for future genetic studies of HCC using A52 cells as a model.

## AUTHOR CONTRIBUTIONS

**Rhys Gillman:** Conceptualization; data curation; formal analysis; funding acquisition; investigation; methodology; software; validation; writing – original draft. **Eun Jin Sun:** Investigation; resources. **Miriam Wankell:** Data curation; formal analysis; resources. **Matt A. Field:** Supervision; validation; writing – review and editing. **Ulf Schmitz:** Supervision; validation; writing – review and editing. **Lionel Hebbard:** Funding acquisition; investigation; project administration; supervision; validation; writing – review and editing.

## FUNDING INFORMATION

This work was supported by Tour De Cure [RSP‐379‐FY2023 to R.G.]; James Cook University Postgraduate Research Scholarship (to R.G.); Tropical Australian Academic Health Centre Limited—Research Seed Grant [SF000121 to L.H.]. National Health and Medical Research Council Investigator Grants (#5121190, MF; #1196405, U.S.); Cancer Council NSW project grant (RG20‐12, U.S.); and the Townsville Hospital Health Service‐Study Education Research Trust Account, Project and Capacity Building Grants (RPG1 2023 and RCG2 2023, MW, MF, US, LH).

## CONFLICT OF INTEREST STATEMENT

We declare no conflicts of interest in this work.

## ETHICS STATEMENT

Animal experimental protocols were approved by the James Cook University Animal Welfare and Ethics committee (A2983). All experiments were performed in accordance with relevant National Health and Medical Research Council (NHMRC) guidelines and regulations.

## Supporting information


Figure S1.

Data S1.

Table S1.

## Data Availability

Raw and processed data are available online at GSE302860.
