# Supplementary material for: Molecular characterization of the A52 murine hepatocellular carcinoma cell line
Source: Animal Model Exp Med. 2026 Feb 27;9(3):546–57. doi: 10.1002/ame2.70152 (PMC13176094; doi:10.1002/ame2.70152)
Supplement: Supplementary file 1 — Figure S1. [file AME2-9-546-s003.docx]

**Molecular Characterisation of the A52 Murine Hepatocellular Carcinoma Cell Line - Supplementary Figures**


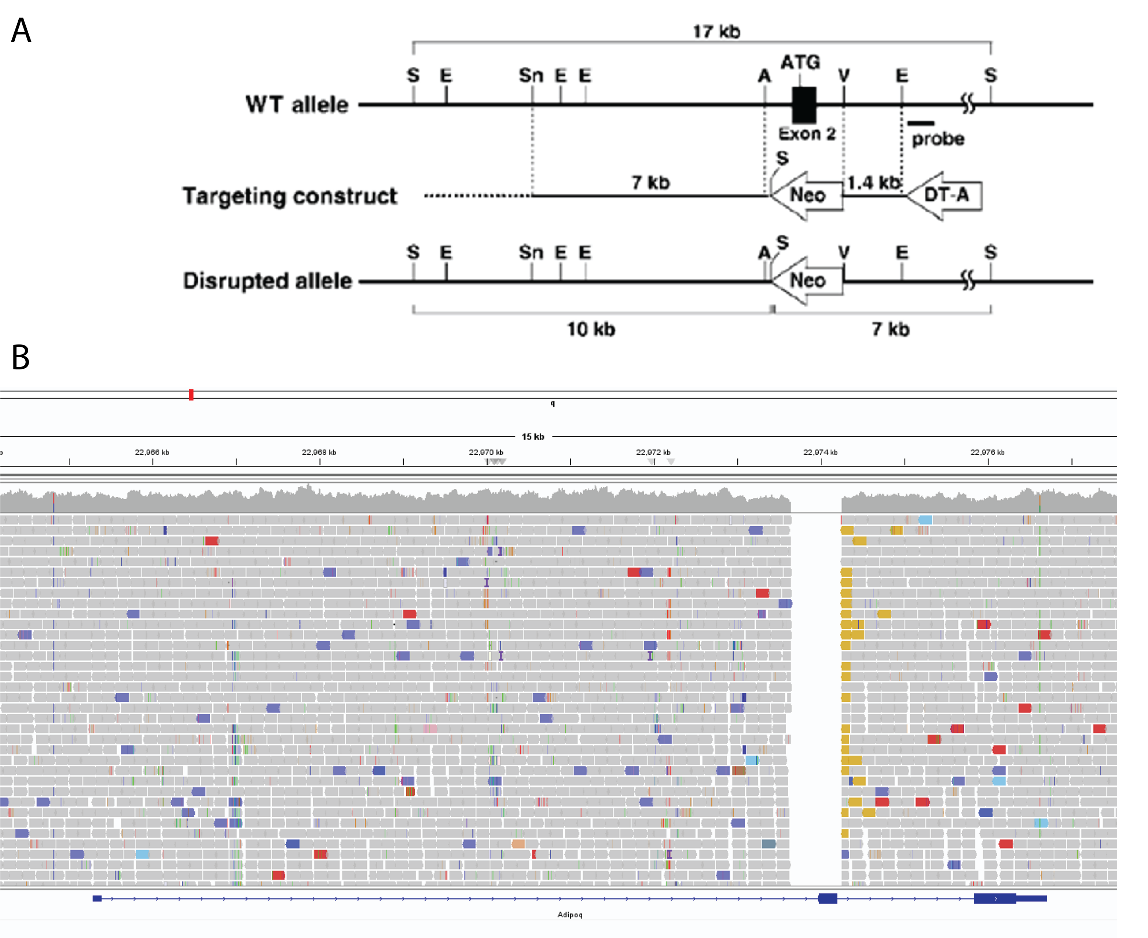


**Supplementary Figure S1: Evidence of Adipoq-knockout in A52.** (A) Figure retrieved from Maeda, Shimomura (1) showing the original knockout *Adipoq*-knockout strategy in C57B/6 mice by replacing the start-codon containing exon2 with a Neomycin (Neo) resistance gene. (B) Successful knockout was confirmed by a sequencing coverage gap in exon 2 of the *Adipoq* gene in whole genome sequencing (WGS) data from A52.


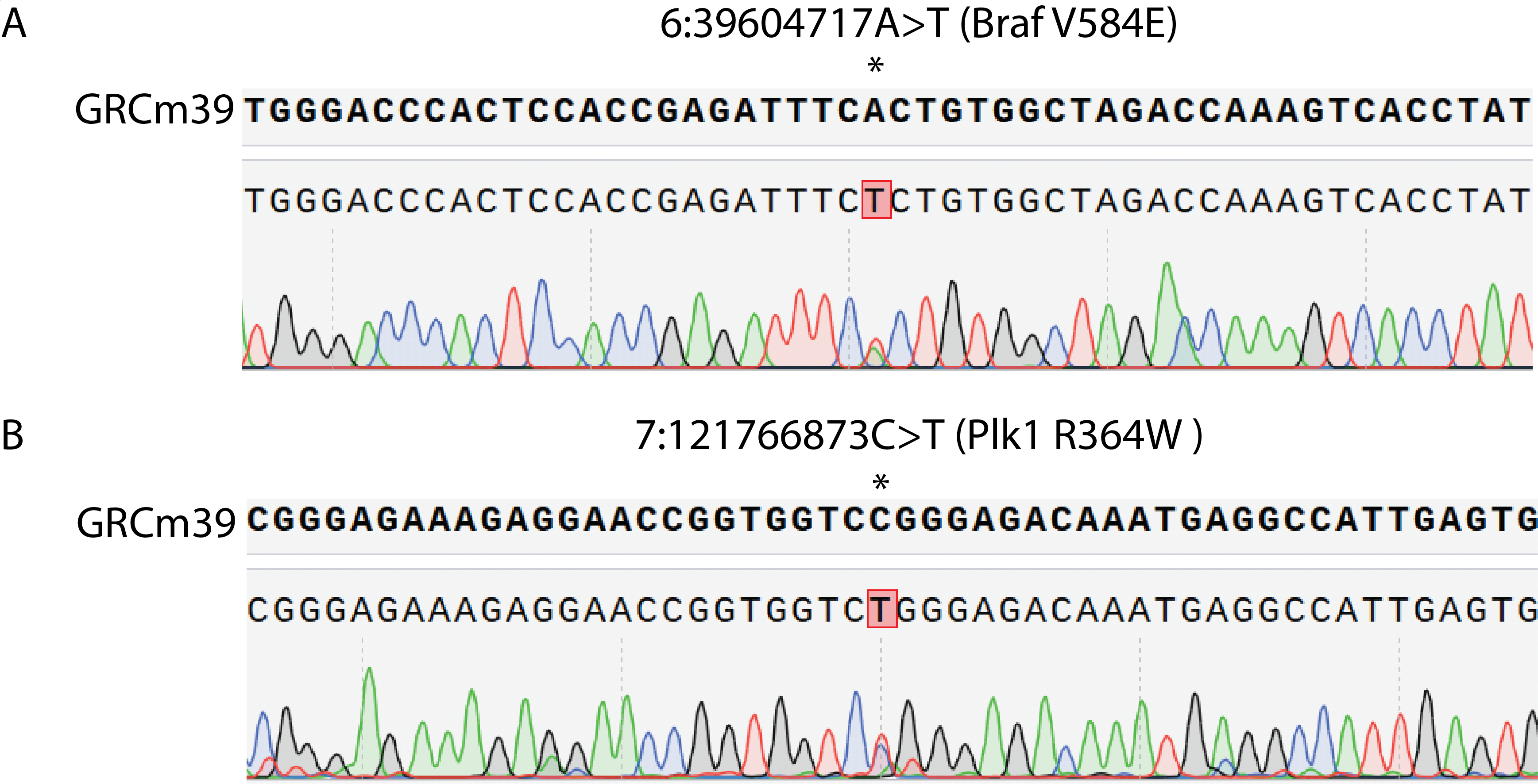


**Supplementary Figure S2: Sanger sequencing confirmation of *Braf* V584E and *Plk1* R364W variants.** Sanger sequencing was performed on amplified regions of the A52 genome surrounding the *Braf* V584E (A) and *Plk1* R364W (B) variants. Trace data indicates the presence of heterozygous single nucleotide substitutions at each of the indicated locations.


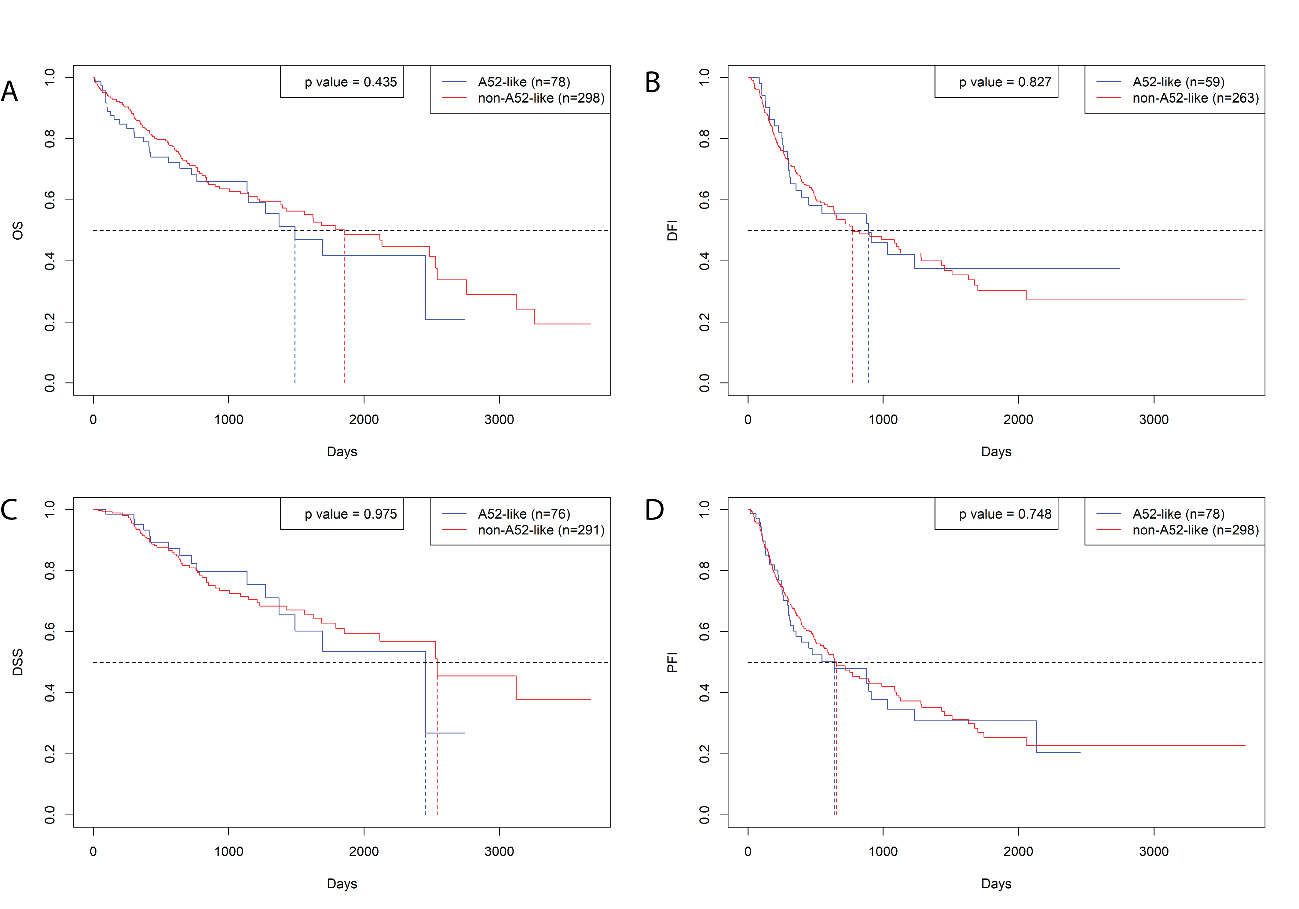


**Supplementary Figure S3: Survival analysis of A52-Like and non A52-Like patients in The Cancer Genome Atlas (TCGA) Liver Hepatocellular Carcinoma (LIHC) cohort.** Patients were divided into two cohorts using hierarchical clustering based on their expression of genes in the A52 signature, as in Figure 5. These patients were compared in terms of: A) overall survival (OS), B) Disease-free interval (DFI), C) Disease-Specific Survival (DSS), and D) Progression-Free Interval (PFI). Comparisons show two-sides p-values for the Log-Rank test using the Chi-Square statistic.

**References**

1. Maeda N, Shimomura I, Kishida K, Nishizawa H, Matsuda M, Nagaretani H, et al. Diet-induced insulin resistance in mice lacking adiponectin/ACRP30. Nat Med. 2002;8(7):731-7.
