# Supplementary material for: Molecular characterization of the A52 murine hepatocellular carcinoma cell line
Source: Animal Model Exp Med. 2026 Feb 27;9(3):546–57. doi: 10.1002/ame2.70152 (PMC13176094; doi:10.1002/ame2.70152)
Supplement: Supplementary file 2 — Data S1. [file AME2-9-546-s001.docx]

**Molecular Characterisation of the A52 Murine Hepatocellular Carcinoma Cell Line - Supplementary Methods**

*Nucleic Acid Extraction and Sequencing*

Genomic DNA (gDNA) was extracted from 1.5 million A52 cells using the Monarch gDNA Extraction Kit (T3010G, New England Biolabs) following the manufacturer's recommended protocol, and eluted in 10mM Tris-HCl (pH 8.8). Extracted gDNA quality was verified by agarose gel electrophoresis (1% gel), and quantified using Qubit Fluorometer with a dsDNA HS Assay Kit (Q33230, Invitrogen). The whole-genome sequencing (WGS) library was prepared, and paired-end sequencing (150 bp reads) conducted on an Illumina NovaSeq platform by the Australian Genome Research Facility (AGRF), achieving an average depth of 100x.

Total RNA was extracted in duplicate from cell pellets of A52 and AML-12 cells, and from five independent tissue samples of subcutaneous A52 tumours, using the Isolate II RNA Mini Kit (BIO-52073, Meridian Bioscience), according to manufacturer guidelines. RNA quality was assessed on an Agilent 2200 TapeStation. RNA Integrity Numbers (RIN) ranged from 8.2-9.1 for tumour tissues and from 9.8–10.0 for cell lines. RNA sequencing libraries were prepared by AGRF and subjected to paired-end sequencing (150 bp reads) on an Illumina NovaSeq platform.

*WGS Data Analysis*

Raw FASTQ sequencing files were retrieved from AGRF and processed according to Genome Analysis Toolkit (GATK) (1) v4.5.0.0 best practices for variant discovery. Briefly, an unmapped bam file was generated using Picard Tools (v3.1.1). Adapter sequences were identified and marked, followed by alignment to the *Mus musculus* reference genome (Ensembl, GRCm39) using Burrows-Wheeler Aligner (BWA, v0.7.17). Duplicate reads were marked using Picard MarkDuplicates. Base Quality Score Recalibration (BQSR) was performed utilising known variant sites from the Sanger Mouse Genome Project (Release 2112, v8, <https://www.mousegenomes.org/snps-indels/>). Small variants were called using HaplotypeCaller, followed by hard-filtering, based on quality metrics and functional annotation using ANNOVAR (version 2020-06-08) (2). Based on these variants, SigProfilerAssignmentR (3) was used to identify tri-nucleotide context mutational signatures and assign variants to a known catalogue of somatic variants in cancer (COSMIC) signatures (4).

A genome-wide read depth analysis was performed using the Samtools (v.1.20) (5) bedcov command in 10kb windows across the entire genome and calculating the mean depth per base for each window. Additionally, structural variants (SVs) were identified with Manta (v1.6.0) (6). For Manta SVs, paired and split read support were combined for the ref and alt alleles to calculate a mutant allele frequency, and only variants with alternate allele frequency > 0.33 were retained.

*Sanger Sequencing*

To confirm the presence of *Braf* V584E and *Plk1* R364W variants, PCR amplification was performed on small genomic regions surrounding the variants from A52 genomic DNA. Sanger sequencing of the resulting PCR amplicons was performed by AGRF. Sequencing traces were aligned to GRCm39 and traces were visualised using SnapGene (v8.2.1).

*RNA-seq Data Analysis*

FASTQ sequencing files from A52 tumour tissue, A52 cells and AML-12 cells were generated by AGRF. Additionally, FASTQ files for three healthy mouse liver samples were retrieved from the RNA mouse atlas (7). Quality control was performed using FastQC (v0.12.1) and summarized with MultiQC (v1.18). Adapter sequences were trimmed using Trimmomatic (v0.39) (8), followed by alignment to the mouse reference genome (Ensembl, GRCm39) using Bowtie 2 (v2.5.4) (9). Gene expression was quantified using RSEM (v1.3.3) (10). Additionally, RSEM count matrices for 377 human HCC tissue samples and 50 non-cancer control samples were retrieved from The Cancer Genome Atlas (TCGA) via the BROAD Institute’s GDAC Firehose portal (<https://gdac.broadinstitute.org/>).

Differential gene expression analysis and variance-stabilizing transformations (VST) were performed with DESeq2 (v1.42.1) (11), using thresholds of adjusted p-value < 0.01 and log2 fold-change > 1 for significance. Pairwise analyses were performed between cancer cell (A52) and normal cell (AML-12), cancer tissue and normal tissue, and cancer cell and cancer tissue. VST expression data was used for principal component analysis and centered to the mean VST of each gene for heatmap visualizations. Heatmaps and gene ontology (GO) analyses utilized ComplexHeatmap (v2.18.0) (12) and TopGO (v2.54.0) packages in R, respectively.

*Statistical Analysis of TCGA Clinical Data*

To identify differences between A52-like and non-A52-like patients in the TCGA cohort, we performed Chi-Square tests with Benjamini-Hochberg (BH) adjustment. Clinical data and variant data were both sourced from XenaBrowser (<https://xenabrowser.net/>). Mutations investigated were restricted to genes mutated in >5% of the TCGA cohort.

*Driver Gene Prediction*

DawnRank (13) was used for *de novo* prediction of driver genes in A52, using the TARGET-SL framework (14). Specifically, we used the DESeq2 variance-stabilising transformed (VST) values for gene expression, and combined the gene expression of all genes across the A52 replicates by their mean. We supplied this and the VST expression of the AML12 samples to the DawnNormalize function, which produces a normalised expression matrix. We also created a binary mutation matrix of genes with non-silent mutations. Finally, we generated a gene interaction network (GIN) using the mouse STRINGdb v11 (15) network filtered to edges with confidence > 0.4 known directionality only. The expression and mutation matrices and GIN were then supplied to the Dawn function to produce a rank-ordered list of driver predictions.

**References**

1. McKenna A, Hanna M, Banks E, Sivachenko A, Cibulskis K, Kernytsky A, et al. The Genome Analysis Toolkit: a MapReduce framework for analyzing next-generation DNA sequencing data. Genome Res. 2010;20(9):1297-303.

2. Wang K, Li M, Hakonarson H. ANNOVAR: functional annotation of genetic variants from high-throughput sequencing data. Nucleic acids research. 2010;38(16):e164.

3. Diaz-Gay M, Vangara R, Barnes M, Wang X, Islam SMA, Vermes I, et al. Assigning mutational signatures to individual samples and individual somatic mutations with SigProfilerAssignment. bioRxiv. 2023.

4. Alexandrov LB, Kim J, Haradhvala NJ, Huang MN, Tian Ng AW, Wu Y, et al. The repertoire of mutational signatures in human cancer. Nature. 2020;578(7793):94-101.

5. Danecek P, Bonfield JK, Liddle J, Marshall J, Ohan V, Pollard MO, et al. Twelve years of SAMtools and BCFtools. Gigascience. 2021;10(2):giab008.

6. Chen X, Schulz-Trieglaff O, Shaw R, Barnes B, Schlesinger F, Kallberg M, et al. Manta: rapid detection of structural variants and indels for germline and cancer sequencing applications. Bioinformatics (Oxford, England). 2016;32(8):1220-2.

7. Sollner JF, Leparc G, Hildebrandt T, Klein H, Thomas L, Stupka E, et al. An RNA-Seq atlas of gene expression in mouse and rat normal tissues. Scientific data. 2017;4(1):170185.

8. Bolger AM, Lohse M, Usadel B. Trimmomatic: a flexible trimmer for Illumina sequence data. Bioinformatics (Oxford, England). 2014;30(15):2114-20.

9. Langmead B, Salzberg SL. Fast gapped-read alignment with Bowtie 2. Nature Methods. 2012;9(4):357-9.

10. Li B, Dewey CN. RSEM: accurate transcript quantification from RNA-Seq data with or without a reference genome. BMC bioinformatics. 2011;12(1):323.

11. Love MI, Huber W, Anders S. Moderated estimation of fold change and dispersion for RNA-seq data with DESeq2. Genome biology. 2014;15(12):550.

12. Gu Z, Eils R, Schlesner M. Complex heatmaps reveal patterns and correlations in multidimensional genomic data. Bioinformatics (Oxford, England). 2016;32(18):2847-9.

13. Hou JP, Ma J. DawnRank: discovering personalized driver genes in cancer. Genome Med. 2014;6(7):56.

14. Gillman R, Field MA, Schmitz U, Hebbard L. TARGET-SL: precision essential gene prediction using driver prioritisation and synthetic lethality. Brief Bioinform. 2025;26(3).

15. Szklarczyk D, Gable AL, Lyon D, Junge A, Wyder S, Huerta-Cepas J, et al. STRING v11: protein-protein association networks with increased coverage, supporting functional discovery in genome-wide experimental datasets. Nucleic acids research. 2019;47(D1):D607-D13.
